# Ex vivo visualization of the trigeminal pathways in the human brainstem using 11.7T diffusion MRI combined with microscopy polarized light imaging

**DOI:** 10.1007/s00429-018-1767-1

**Published:** 2018-10-06

**Authors:** Dylan J. H. A. Henssen, Jeroen Mollink, Erkan Kurt, Robert van Dongen, Ronald H. M. A. Bartels, David Gräβel, Tamas Kozicz, Markus Axer, Anne-Marie Van Cappellen van Walsum

**Affiliations:** 1 Department of Anatomy, Donders Institute for Brain, Cognition and Behavior, Radboud University Medical Center, Geert Grooteplein Noord 21, 6525 EZ Nijmegen, The Netherlands; 2 Department of Neurosurgery, Radboud University Medical Center, Nijmegen, The Netherlands; 3 Department of Clinical Neurosciences, Wellcome Centre for Integrative Neuroimaging, FMRIB, University of Oxford, Oxford, Nuffield UK; 4 Department of Anesthesiology, Pain and Palliative Care, Radboud University Medical Center, Nijmegen, The Netherlands; 5 Institute of Neuroscience and Medicine (INM-1), Research Centre Jülich, Jülich, Germany; 6 Department of Clinical Genomics and Biochemistry and Molecular Biology, Mayo Clinic, Rochester, USA

**Keywords:** Brainstem, Trigeminal pathways, Anatomy, Histology, Ex-vivo high-resolution imaging, MRI, Diffusion-weighted imaging, Polarized light imaging

## Abstract

Classic anatomical atlases depict a contralateral hemispheral representation of each side of the face. Recently, however, a bilateral projection of each hemiface was hypothesized, based on animal studies that showed the coexistence of an additional trigeminothalamic tract sprouting from the trigeminal principal sensory nucleus that ascends ipsilaterally. This study aims to provide an anatomical substrate for the hypothesized bilateral projection. Three post-mortem human brainstems were scanned for anatomical and diffusion magnetic resonance imaging at 11.7T. The trigeminal tracts were delineated in each brainstem using track density imaging (TDI) and tractography. To evaluate the reconstructed tracts, the same brainstems were sectioned for polarized light imaging (PLI). Anatomical 11.7T MRI shows a dispersion of the trigeminal tract (*tt*) into a ventral and dorsal portion. This bifurcation was also seen on the TDI maps, tractography results and PLI images of all three specimens. Referring to a similar anatomic feature in primate brains, the dorsal and ventral tracts were named the dorsal and ventral trigeminothalamic tract (*dtt* and *vtt*), respectively. This study shows that both the *dtt* and *vtt* are present in humans, indicating that each hemiface has a bilateral projection, although the functional relevance of these tracts cannot be determined by the present anatomical study. If both tracts convey noxious stimuli, this could open up new insights into and treatments for orofacial pain in patients.

## Introduction

**Fig. 1:**
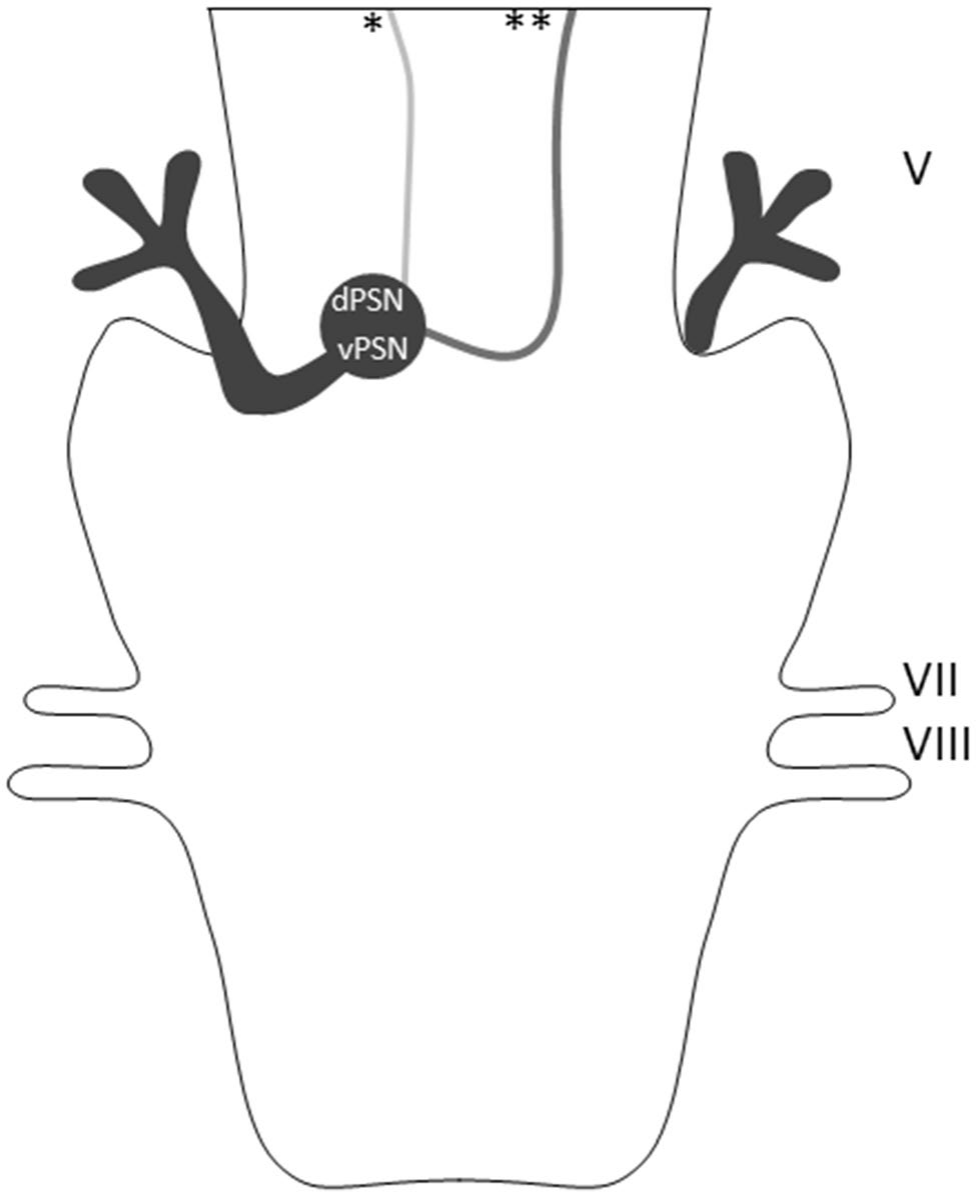
Overview of the trigeminothalamic tracts that arise from the PSN in a dorsal view. Cranial nerves indicated in Roman numerals; *dPSN* dorsal principal sensory nucleus; *vPSN* ventral principal sensory nucleus; *dorsal trigeminothalamic tract (*dtt*), **ventral trigeminothalamic tract (*vtt*)

Classically, orofacial nociceptive afferents, running through the trigeminal nerve and the trigeminal tract (TT), are believed to synapse in the ipsilateral trigeminal sensory nucleus complex (Sessle 2000). Second-order neurons then cross and ascend as the trigeminothalamic tract to the contralateral ventro-posterior medial nucleus of the thalamus. From there, projections ascend to the primary and secondary somatosensory cortices and other cortical regions, such as the insula (Ralston 2005). However, the exact anatomy of the central portion of the trigeminal pathways in humans remains a point of discussion. Knowledge of the exact neuroanatomy of the trigeminal tracts will contribute to the understanding of orofacial pain and its treatment. Preclinical experiments provide evidence for a bilateral projection of the orofacial region causing activation of both thalami and both primary sensory cortices in healthy subjects, as well as patients suffering from chronic orofacial pain (Nash et al. 2009, 2010). This bilateral representation of the orofacial region can be explained by transcallosal pathways or by a bilateral trigeminothalamic system. The involvement of transcallosal pathways in the bilateral activation pattern of the aforementioned brain regions in the registration of orofacial pain seems to be of limited importance according to the split-brain study of Stein et al. However, proper interpretation of the results of this study is hampered due to the small sample size (*n* = 1) (Stein et al. 1989). Additionally, Solstrand Dahlberg and colleagues report findings that a decreased connectivity of regions in the brainstem that are involved in orofacial pain with parts of the descending pain modulation system is present in patients suffering from migraine. However, they also conclude that the exact anatomy of the trigeminal pathways in the human brainstem remain elusive (Solstrand Dahlberg et al. 2018). In 2016, a review of tracing studies in animals and functional MRI studies in humans provided an overview of the trigeminal tracts in the brainstem (Henssen et al. 2016). Figure 1 depicts two trigeminothalamic tracts sprouting from the principal sensory nucleus (PSN), the ventral- and dorsal trigeminothalamic tract (*vtt* and *dtt*, respectively). The *vtt* is a mainly contralateral pathway and is known to be present in human specimens. The *dtt*, on the other hand, is a mainly ipsilateral pathway and is predominantly known from animal-based studies and has only been hypothesized to exist in humans (Henssen et al. 2018).

Based on the aforementioned results, we hypothesized that *dtt* would exist in the human brainstem too. To test this hypothesis, we investigated the neuroanatomy of the *dtt* in human brainstems using, multiple, advanced neuroimaging techniques, including post-mortem, 11.7 anatomical magnetic resonance imaging (MRI) and diffusion-weighted MRI (dMRI) and several post-processing techniques (e.g., tract density imaging (TDI) and tractography). To complement the MR findings, the same brainstems were sectioned to obtain histological transverse sections for polarized light imaging microscopy (PLI). PLI is a microscopy technique to quantify fiber orientation based on birefringence of the myelin sheath in histological brain sections and has been reported as a well-suited technique to validate MR findings (Axer et al. 2011b).

## Materials and methods

### Acquisition of the post-mortem material

Three post-mortem brains were derived from the body donor program of the department of Anatomy at Radboud University Medical Center, Nijmegen, The Netherlands. The three donors had no known neurological diseases and none of the brains showed pathological deformities after death. All specimens were perfused via the femoral artery using 10% formaldehyde and a relatively short postmortem interval (< 24 h) was employed to limit the reduction of several diffusion values [apparent diffusion coefficient (ADC) and fractional anisotropy (FA)] (D’Arceuil and de Crespigny 2007; Schmierer et al. 2008). After embalmment, the specimens were fixated in 7.7% formaldehyde for at least 3 months to a maximum of 3 years as it was suggested that diffusivity measures remain stable up to 3 years after fixation (Dyrby et al. 2007). Then the brain was extracted from the skull. The brainstem and cerebellum were separated from the cerebrum via a transverse section, perpendicular to the neural axis, at the level of the superior colliculus. The cerebellum was dissected from the brainstem by a coronal cut through the middle cerebellar peduncle. All three parts were fixated in 7.7% formaldehyde and preserved for another 2 months. See Table 1 for additional information. This study was carried out in accordance with the recommendations of the CMO (Commissie Mensgebonden Onderzoek) region Arnhem–Nijmegen, Netherlands. The post-mortem specimens were acquired via the body donor program at the department of Anatomy of the Radboud university medical centre, Nijmegen, Netherlands. All body donors in this program signed a written informed consent during their lifetime, permitting the use of their body and parts for science and teaching.

**Table 1:** Overview of the characteristics of the specimens used in this study

Characteristics	Subject 1	Subject 2	Subject 3
Age (years)	62	77	76
Gender	Female	Male	Female
Cause of death	Colon cancer	Pneumonia	Colon cancer/euthanasia
Time between death and fixation (h)	12	17	23
Time between fixation and extraction of the brain (months)	3	2	2
Time between extraction of the brain and scanning (months)	2	2	2

### Magnetic resonance image acquisition

Prior to MR scanning, the three post-mortem brainstems were stored in a phosphate-buffered saline solution (PBS 0.1M, pH 7.4) for 1 week to reverse the decrease of the T2 relaxation rate of tissue introduced by formaldehyde (Shepherd et al. 2009). Next, the brainstems were placed for 24 h in Fomblin® (*Solvay Solexis Inc*), a susceptibility-matched, hydrogen-free liquid. The brainstems were then placed in a vacuum chamber for 10 min to reduce free air in the lumina of the blood vessels. Ultimate maximum pressure within the vacuum chamber was set to 1350 mmHg to prevent cell membrane damages (Gonzalez-Rodriguez et al. 2016; Holzapfel et al. 2005), although the maximum pressure did not exceed 500 mmHg during the experiments as this was not necessary to remove air bubbles. Finally, each brainstem was placed in a 100 ml syringe, filled with Fomblin, for MR scanning. All imaging was performed on a 11.7T Bruker BioSpec Avance III preclinical MR system (Bruker BioSpin, Ettlingen, Germany) equipped with an actively shielded gradient set of 600 mT/m (slew rate 4570 T/m/s). A circular polarized resonator was used for signal transmission and an actively decoupled birdcage coil (Bruker Biospin) was used for receiving. Scanning was performed at room temperature (20 °C). T2* weighted images were acquired using a 3D multi-gradient echo sequence at 0.25 mm isotropic resolution with the following parameters: TR = 3314 ms; TE = 7 ms; and flip angle = 20°. The dMRI data were obtained using segmented spin-echo echo-planar imaging at 0.5 mm isotropic resolution. This sequence consisted out of four segments. A total of 256 gradient directions were employed at a *b* value of 4000 s/mm^2^, in addition to six images with no diffusion weighting (*b* = 0 s/mm^2^) and the following gradient and pulse parameters: *Δ* = 12.5 ms, *δ* = 4.0 ms, TE = 30.70 ms, and TR = 13.75 s. The applied MR protocol was adapted from an empirically designed protocol reported in the literature (Kleinnijenhuis et al. 2013).

### Probabilistic tractography

All processing of the MR images was performed with FSL (Jenkinson et al. 2012). To correct for eddy current artifacts and displacement between the different diffusion images, an eddy current correction was applied (Andersson and Sotiropoulos 2015). The dMRI data were pre-processed for tractography with the bedpostX algorithm that models multiple fiber orientations (*n* = 3) at each voxel (Behrens et al. 2007). To delineate the TT in our specimens, probabilistic tractography was performed using the Probtrackx2 algorithm. A manually defined seed mask (i.e., the starting point of tractography) was placed in the entry zone of the trigeminal nerve on the fractional anisotropy (FA) maps. To address the reproducibility of the drawing of the masks in each specimen, two neuroanatomists, independently, delineated the location of the trigeminal root entry zone on both sides of each brainstem. The overlap of both masks was used as the seeding masks for tractography on either the right or left side of each brainstem. Streamlines were drawn from each seed-voxel (*n* = 50,000 streamlines per voxel). Only streamlines that reached the dorsal part of the brainstem near the PSN—defined by a waypoint mask—were kept to avoid the inclusion of other large dominating white tracts that run through the brainstem (Büttner-Ennever and Horn 2013). Maximum intensity projections (MIPs) of the trigeminal tract streamlines along aspecified encoded color direction, which improve the visualization of general tractography results as they provide stereoscopic visualization, were overlaid on the fractional anisotropy, which highlights the tract locations.

### Track density imaging

Track density imaging (TDI) is typically used to investigate white matter microstructure smaller than the imaging resolution by reinterpolation of the quantitative maps by constrained spherical deconvolution based on the response function at a finer resolution (Calamante et al. 2010). TDI was applied to the dMRI data to study the TT trajectory and its possible bilateral projections. To generate the directionally encoded color TDI maps, whole brainstem probabilistic fiber-tracking was carried out with MRtrix as described by Calamante et al. (2010). Streamlines were generated (total number of streamlines: *n* = 500,000; 20 per seed voxel) from a large number of random seeds throughout the brainstem. Seeds are placed randomly throughout the brainstem as described by other before (Calamante et al. 2010, 2011). The total number of streamlines was calculated in each element of a grid that covers the brainstem. It should be noted that the sampling distance of the grid was smaller than the acquired voxel size, yielding TDI map with higher spatial resolution than the source dMRI data (0.1 mm isotropic). After tracking, the streamline density was used as intra-voxel information to construct a super-resolution TDI image (Calamante et al. 2010).

### Histological tissue processing

Histological sectioning was performed for the TT at the level of the trigeminal nerve entry zone. This part was separated from the rest of the brainstem with a transverse cut through the inferior olivary nucleus and the inferior colliculus. To prevent the formation of ice crystals during freezing, the brainstems were stored in a 30% sucrose PBS solution (PBS 0.1M, pH 7.4) at 4 °C for 1 week. The specimen was frozen using dry ice and serially sectioned with a HM 450 Sliding Microtome (*Thermo Fisher Scientific Inc., Waltham, Massachusetts, USA*) at a thickness of 100 microns. In a series of five axial sections, the first section was mounted on glass, creating an inter-slice gap of 500 microns.

### Polarized light imaging

PLI utilizes the optical birefringence of nerve fibers, which can be attributed to the arrangement of lipid layers in the myelin sheaths wrapping the axons (Axer et al. 2011a, c). Birefringence alters the polarization state of the light that is passed through the specimen depending on the orientation of the myelinated nerve fibers. As such, taking a series of images while rotating the linear polarizer plane allows for quantification of fiber orientation at each pixel. The abovementioned principle of measurement is referred to as polarimetry. To realize PLI measurements of the transverse sections of the brainstems, two polarimeters were used: a large-area polarimeter (LAP) for single shot medium-resolution (64 microns per pixel) images covering the whole tissue section and a polarization microscope (PM) to scan a grid of high-resolution images (1.3 microns per pixel) in smaller regions of interest. Both types of polarimetric equipment work in a similar manner. Monochrome green light passes through a linear polarizer combined with a quarter wave retarder with its fast-axis oriented at 45° with respect to the polarizer plane. This creates circularly polarized light that passes through the specimen followed by another polarizer, which captures the change in polarization induced by tissue birefringence. By simultaneous rotation of all polarizing filters, the transmission intensity is systematically imaged at discrete filter rotation angles. A typical PLI raw image dataset comprised 18 snapshots at equidistant azimuth angles of 10°, ranging between 0° and 170°. On the LAP these measurements were supplemented by 8° tilt measurements to the cardinal points allowing for stereoscopic imaging to disambiguate the fiber elevation angle. Processing of all raw images created, due to the coarse resolution of the LAP-particular anatomical landmarks of the trigeminal pathways in the brainstem, were scanned additionally in the PM to obtain images of higher resolution. The PM used a reversed light pathway and rotates the polarizer only. A more extensive description of the polarimeters engaged during PLI is given by (Reckfort et al. 2015; Axer et al. 2011a, b).

## Results

### T2* weighted MR images

**Fig. 2:**
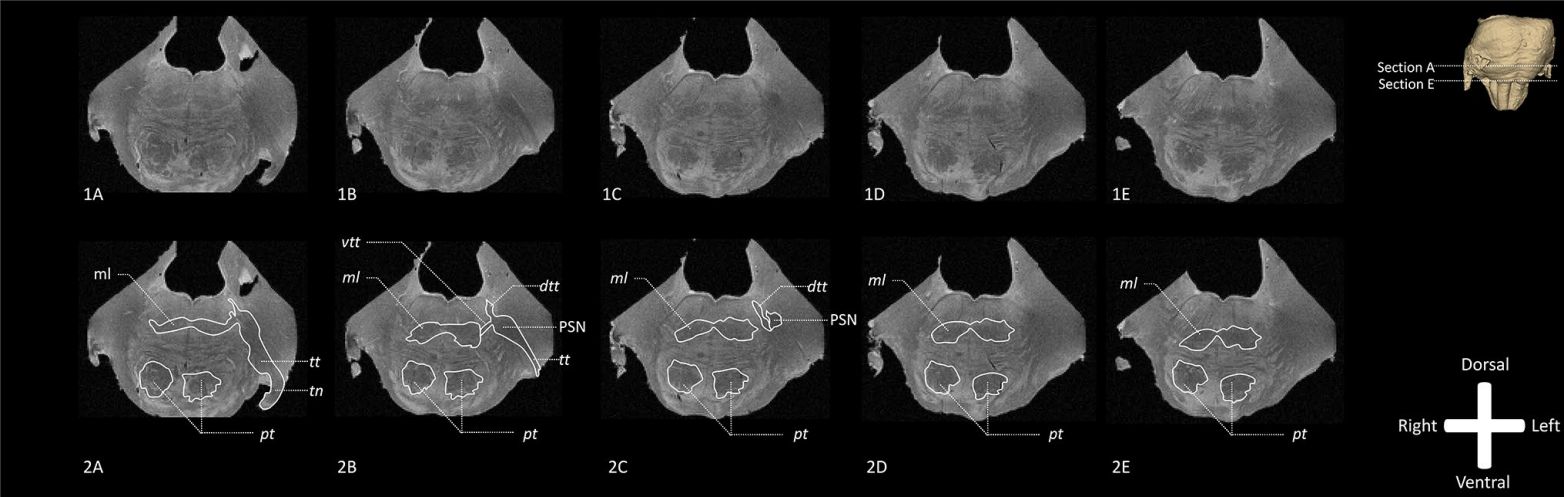
Overview of the transverse T2* weighted MR images of the human brainstem (specimen 2). The TN enters the pons at the ventrolateral part and the *tt* courses in a dorsomedial direction towards the PSN, where it bifurcates into a ventral and a dorsal branch. The ventral branch joins the *ml* whereas the dorsal one propagates towards the mesencephalic nucleus. This configuration has been described in animals as the *vtt* and *dtt. dtt* Dorsal trigeminothalamic tract, *ml* medial lemniscus, *PSN* principal sensory nucleus, *pt* pyramidal tract, *vtt* ventral trigeminothalamic tract, *TN* trigeminal nerve, *tt* trigeminal tract

The trigeminal nerve can be clearly observed entering the ventrolateral part of the pons, after which the hypo-intense signal of the *tt* can be seen traversing the brainstem. The *tt* penetrates the middle cerebellar peduncle (*mcp*) and slopes slightly through the pons towards a large hypo-intense bulge medial to the *mcp*. This enlarged portion of the *tt* represents the PSN (Fig. 2). The PSN is located lateral and dorsal to the medial lemniscus (*ml*) and ventral to the central tegmental tract. At Fig. 2D, two hypo-intense extensions of the PSN, dorsal towards the fourth ventricle (most probably *dtt*) and ventral towards the center of the brainstem (*vtt*), can be discriminated.

### TDI-results

**Fig. 3:**
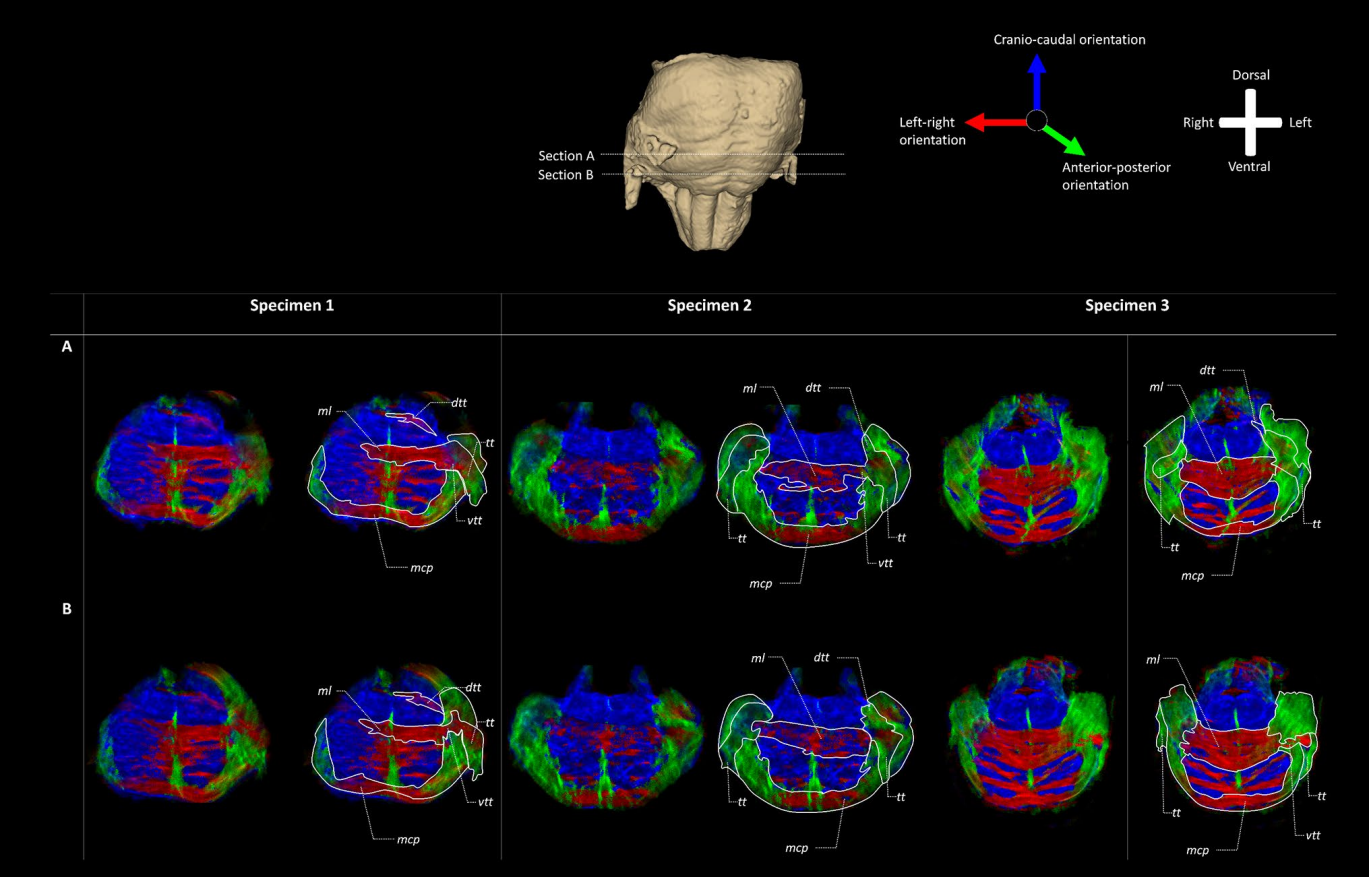
TDI fiber orientation maps of the three brainstems in two representative transverse sections at the trigeminal entry level. The RGB (red–green–blue) color cross indicates the principal eigenvector orientations, red = left–right, green = anterior–posterior, blue = cranial–caudal. The fiber density is encoded by brightness. The fiber orientation is represented by color, while the fiber density is encoded by brightness. The trigeminal tract bifurcates into a ventral and dorsal branch. The ventral branch could be identified as the *vtt* while the dorsal branch corresponds to the *dtt*. The *vtt* turns towards the raphe where it converges partially with the *ml*. The *dtt* courses towards the dorsal wall of the tegmentum where the trigeminal mesencephalic nucleus is located. There, the anterior–posterior orientation changes into a cranio-caudal orientation. *dtt* Dorsal trigeminothalamic tract, *mcp* middle cerebellar peduncle, *ml* medial lemniscus, *tt* trigeminal tract, *vtt* ventral trigeminothalamic tract

**Fig. 4:**
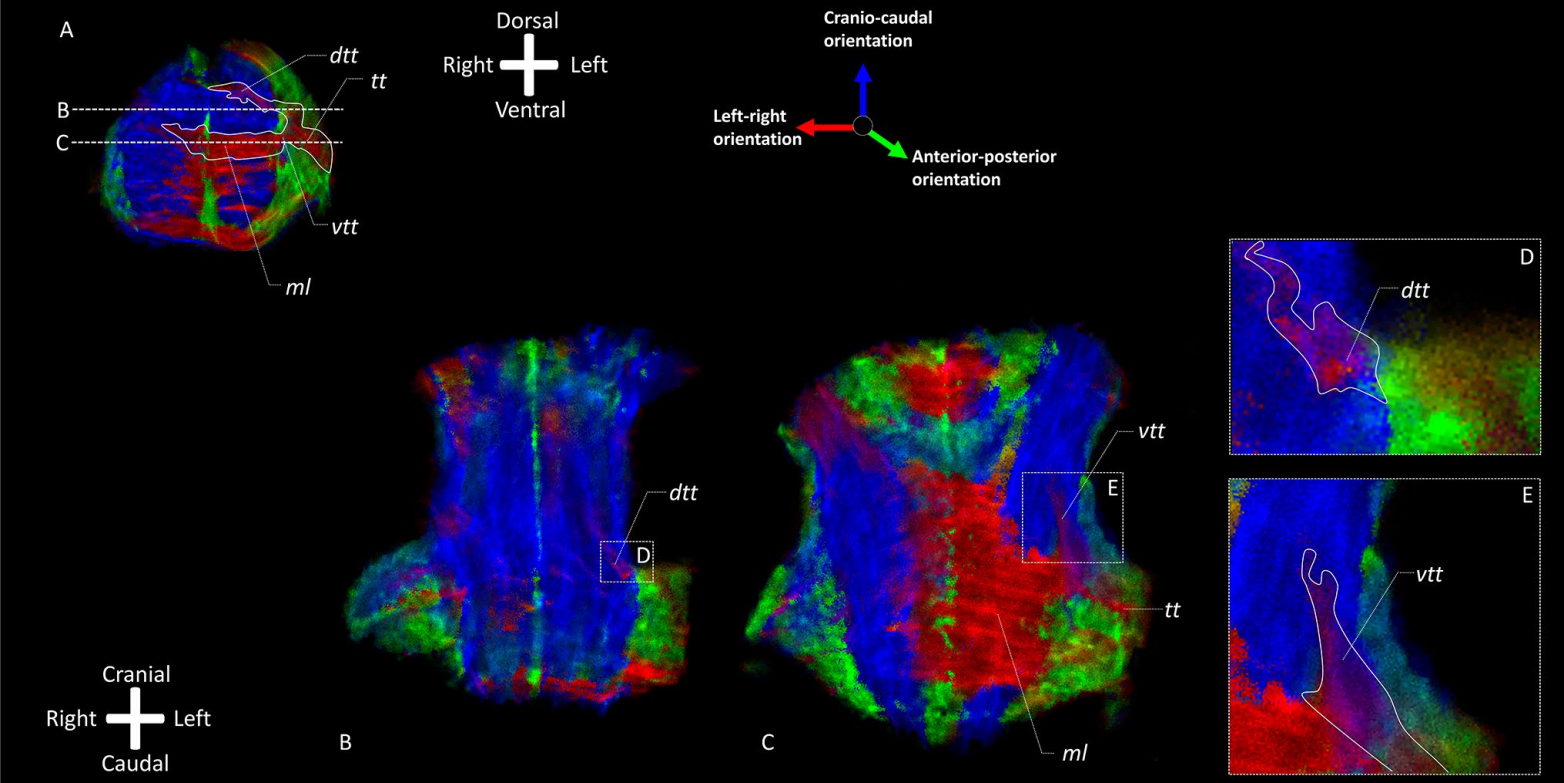
TDI fiber orientation maps of one brainstem (specimen 1) in two directions: transverse sections at the trigeminal entry level and frontal sections at the level of the *vtt* and *dtt*. The RGB (red–green–blue) color cross indicates the principal eigenvector orientations, red = left–right, green = anterior–posterior, blue = cranio-caudal. Insets of **b** and **c** with a higher level of detail are shown in **d** and **e. a** Transverse TDI map of one brainstem (specimen 1) at the level of the entry of the trigeminal nerve. The bifurcation of the trigeminal tract into *vtt* and *dtt* is depicted. Section B is positioned at the level of the *dtt* and section C at the level of the *vtt* as indicated by dashed lines. **b** Frontal TDI map of one brainstem (specimen 1) at the level of the *dtt*. Initially the *tt* has a sagittal orientation after which it sprouts into a transversal. After sprouting from the *tt*, the *dtt* courses towards the dorsal wall of the brainstem. **c** Frontal TDI map of one brainstem (specimen 1) at the level of the *vtt*. The *tt* has a mixed anterior–posterior/left–right orientation. The *vtt* sprouts of from the inner face of the *tt* and attaches itself to the *ml* dorsally where it turns upward to the cranial part of the brainstem. **d** Enlargement of inset D in frontal TDI map depicted in section B. This inset shows the *dtt* in a frontal view and depicts that the *tt* has a anterior–posterior orientation, whereas the *dtt* has a mixed left–right/craniocaudal orientation in the dorsal area of the brainstem. *dtt* Dorsal trigeminothalamic tract, *ml* medial lemniscus, *tt* trigeminal tract, *vtt* ventral trigeminothalamic tract

Figure 3 presents an overview of the results of the TDI analysis of the three specimens in transverse sections confirming the findings of T2* weighted MRI. It can be observed that the *tt* traverses through the *mcp* in a dorsomedial and caudal direction. A portion of it appears to join the *ml*, whereas the rest remains on its dorsomedial course through the tegmentum. After the bifurcation of the *tt*, two tracts can be observed: (1) a dorsomedial tract that courses in cranio-caudal direction and (2) a more ventral tract, dorsal to the *ml*. Figure 4 shows a ventral and a dorsal region of the brainstem as maximum intensity projection of multiple coronal sections near the bifurcation. Their location is shown in (A). The ventral region (C–E) shows that the majority of fibers from the *tt* join the *ml* crossing to the contralateral hemisphere. The dorsal region (B–D) reveals a smaller tract coursing towards the raphe without crossing. Instead it turns into a cranio-caudal direction. In principle, these models reconfirm a bifurcation of the *tt* near the transverse pontine fibers and the *ml* into a dorsal and ventral part. The dorsal tract appears to remain on the ipsilateral side with regard to the trigeminal root from which it sprouts, while the ventral one crosses to the contralateral side of the brainstem. The dorsal tract can, therefore, be determined to be the *dtt*; the ventral one to be the *vtt*.

### Tractography results

**Fig. 5:**
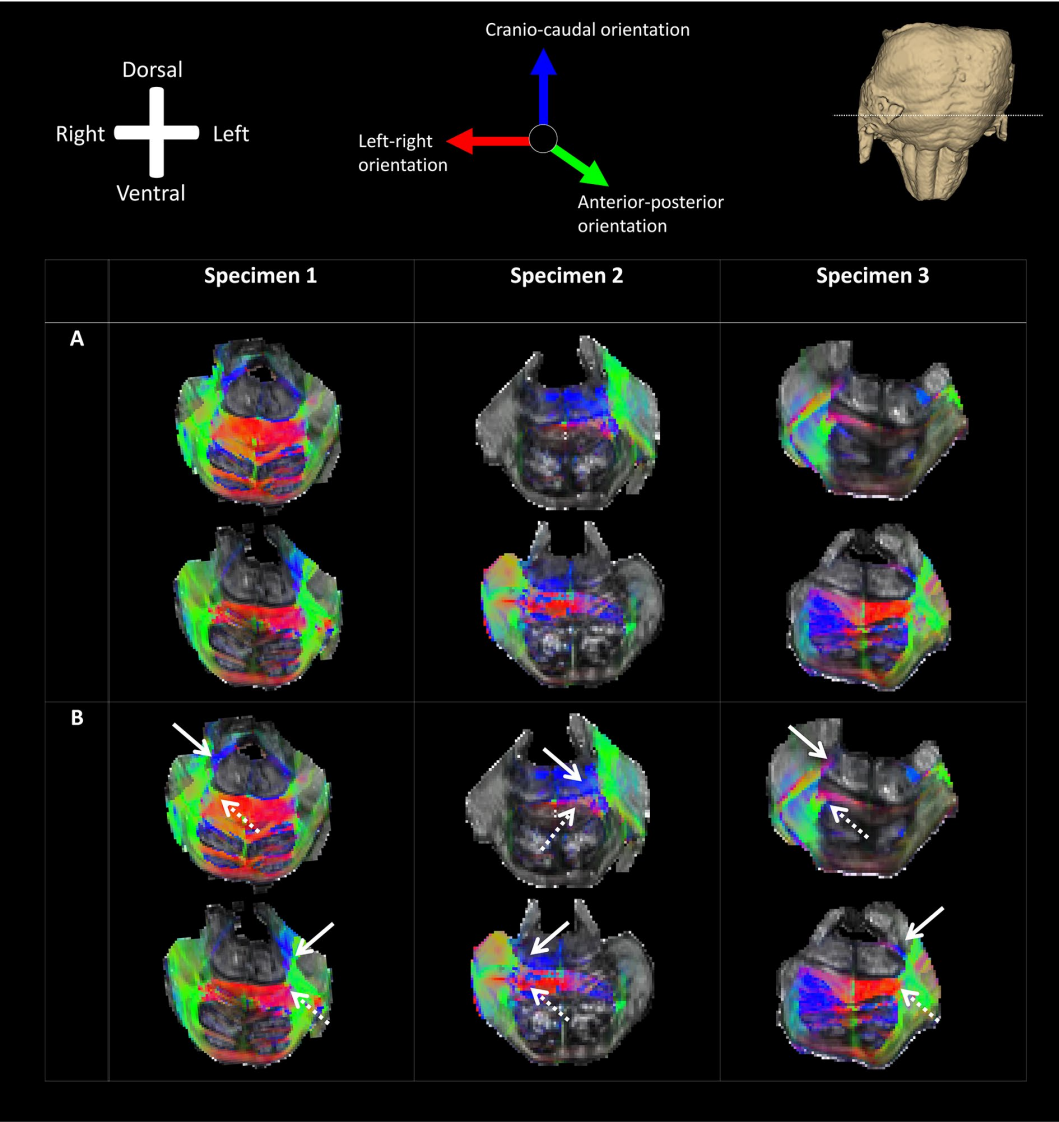
Maximum intensity projection (MIP) of the trigeminal pathways (right = red, left = blue) in all three specimens taken from tractography results of dMRI overlain on 11.7T fractional anisotropy of a transverse section located at the bifurcation of the trigeminal tract. The *tt* can be observed to bifurcate into a ventral and dorsal division, the ventral and dorsal trigeminothalamic tracts respectively. In specimen 3, a third sprout of the right *tt* between the dorsal and ventral division courses towards the cerebellum via the stumb of the middle cerebellar peduncle. The white arrows point at the *dtt*. The white dotted arrows point at the *vtt. dtt* Dorsal trigeminothalamic tract, *tt* trigeminal tract, *vtt* ventral trigeminothalamic tract

Tractography results are depicted stereoscopically by use of MIP images. Results from tractography show a bifurcation of the left (blue) and right *tt* (red) in all three specimens. From this bifurcation, two tracts can be recognized: a dorsomedial one, which courses in cranio-caudal direction remaining ipsilateral, and a ventral one crossing to the contralateral side at the dorsal surface of the *ml*. The dorsal tract can, therefore, be identified as *dtt*, the ventral one as *vtt* (Fig. 5).

### Polarized light images

**Fig. 6:**
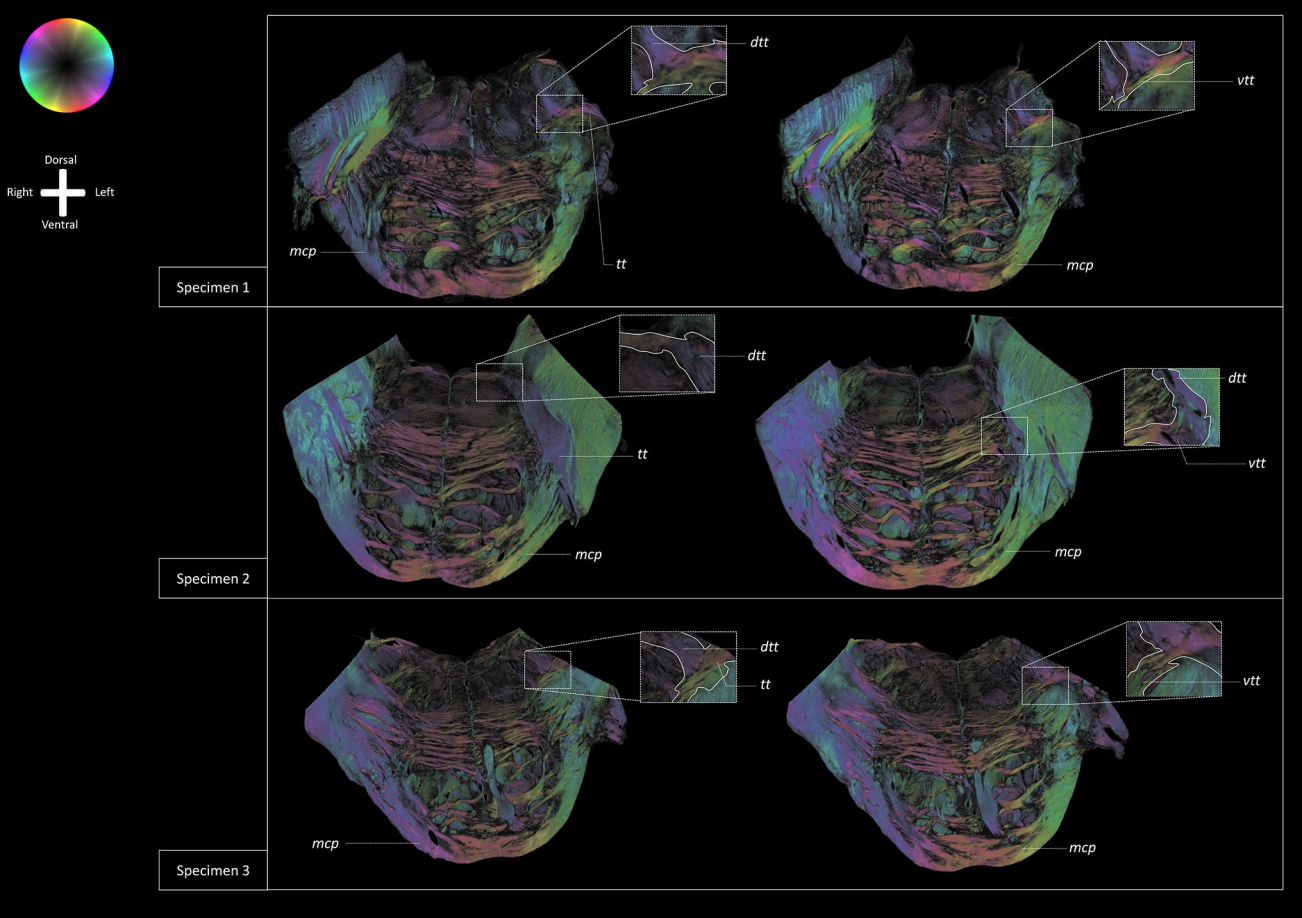
PLI fiber orientation maps of transverse sections of the brainstem of all three specimens at the level of the bifurcation of the trigeminal tract. The fiber orientation is defined by the color sphere. The *tt* obviously penetrates the *mcp* as it courses towards the fourth ventricle. After crossing the *mcp*, a curvature in the course of the *tt* can be observed. Part of the fibers bend towards the dorsal border of the *ml* while the remainder propagates towards the fourth ventricle finally turning in a medial direction towards the raphe of the brainstem. *mcp* Middle cerebellar peduncle, *ml* medial lemniscus, *tt* trigeminal tract, *vtt* ventral trigeminothalamic tract, *dtt* dorsal trigeminothalamic tract

**Fig. 7:**
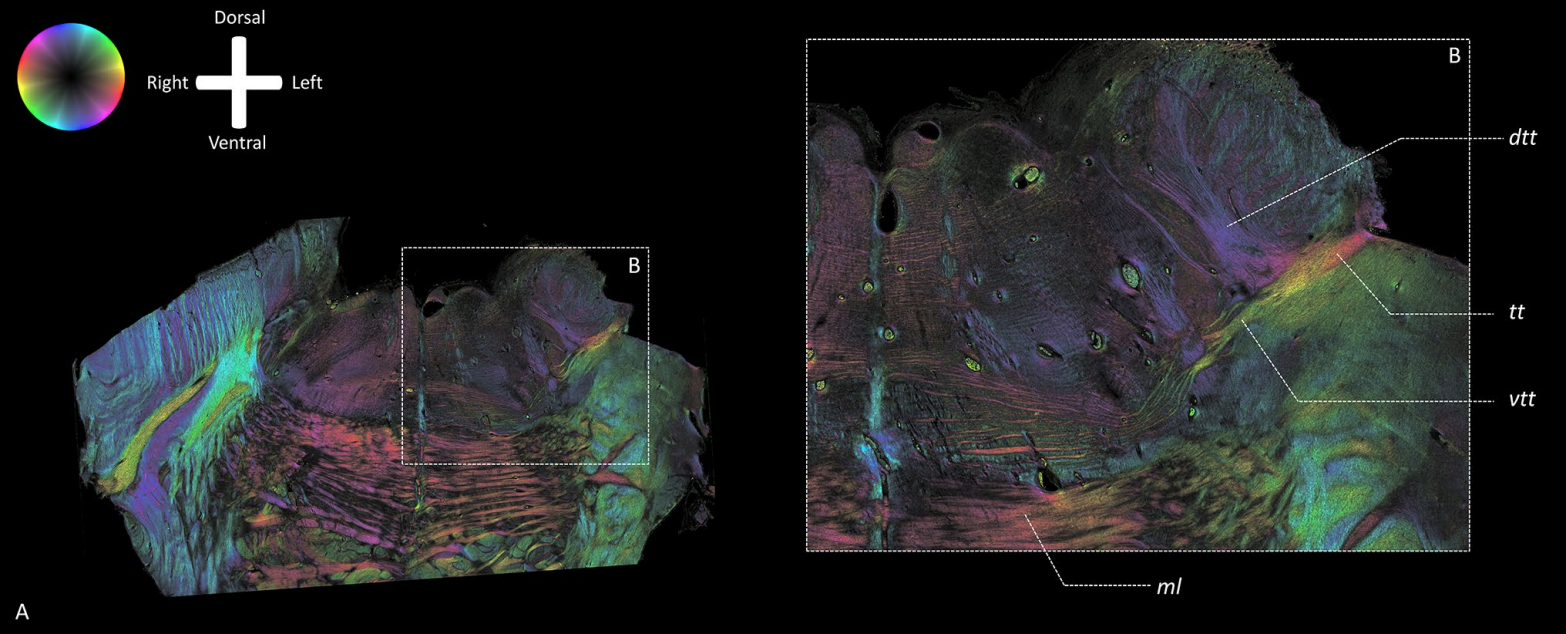
PLI fiber orientation maps acquired at high resolution of transverse sections of the brainstem of specimen 1 at the level of the bifurcation of the trigeminal tract. The fiber orientation is defined by the color sphere in the upper left corner. **a** The *tt* can be observed in the *mcp* (green). The *tt* bifurcates into two tracts: the ventral portion (yellow) and dorsal portion (purple). **b** Detailed view of the bifurcation of the left *tt* shown in **a**. The *vtt* turns alongside the posterior wall of the *ml* and courses towards the raphe of the brainstem. The *dtt* runs towards the dorsal aspect of the brainstem splitting up again towards the cerebellum via the *mcp* (green-aquamarine) and towards the mesencephalic nucleus (purple). *ml* Medial lemniscus, *tt* trigeminal tract, *vtt* ventral trigeminothalamic tract, *dtt* dorsal trigeminothalamic tract

On the PLI images (Fig. 6), the trigeminal root is observed to enter the ventrolateral aspect of the brainstem. The *tt* then crosses the *mcp* after which it bifurcates into a ventral and dorsal division. The ventral division, recognized as the *vtt*, bends alongside the *ml*. The dorsal division, recognized as the *dtt*, runs towards the tegmentum and the fourth ventricle (Fig. 6). Figure 7 depicts the *tt* coursing towards the dorsomedial aspect of the brainstem. After exiting the *mcp*, the *tt* shows a bifurcation into two tracts. The ventral branch runs posterior to the *ml* and courses towards the midline of the brainstem. The dorsal tract runs towards the dorsal aspect of the brainstem. The dorsal tract furthermore shows dispersing fibers that run towards the cerebellum via the *mcp* and towards the dorsomedial area of the brainstem.

## Discussion

For the first time, the existence of a bifurcation of the *tt* into a *vtt* and *dtt* in the human brainstem using (d)MRI, TDI and PLI has been reported. It is well known that the orofacial nociceptive afferents synapse in the ipsilateral trigeminal sensory nuclear complex (TSNC) cross the midline and ascend as the trigeminothalamic tract to the contralateral ventroposterior medial nucleus (VPM) of the thalamus. This study shows that the crossing tract can be identified as the *vtt*. The dorsal branch of the bifurcation could represent the hypothesized ipsilateral *dtt*.

### Comparison with existing studies

In primates, evidence of this bilateral somatosensory pathway through the thalamus to the facial representation of the primary somatosensory cortex has been previously provided (Rausell and Jones 1991a, b). In humans, this bilateral representation pattern has been observed in functional MRI (fMRI) studies that investigated the activation patterns of orofacial pain in humans. These studies showed that unilateral noxious stimulation of the orofacial region evokes changes in both the contralateral and the ipsilateral thalami, insular and primary somatosensory cortices (Jantsch et al. 2005; Nash et al. 2010). Other studies suggested that a bifurcation of the trigeminal nerve can be found at the level of the brainstem, based on the blood oxygen level dependent (BOLD) activation patterns in experimental orofacial pain settings (Nash et al. 2009, 2010), which is consistent with the results of this paper. Furthermore, it is known from developmental brain research that the raphe of the brainstem is a generator of attractants and repellents that channel axonal growth to the proper side of the central nervous system, which causes the crossing of the major somatosensory and motor pathways. However, embryological development studies in mice have shown that a minority of the axons that sprout from the PSN do not cross the midline (Kivrak and Erzurumlu 2013).

The *vtt* has been described to consist of fibers that sprout from the ventral part of the *PSN* and the cranial two-thirds of the spinal nucleus (SN). The axons of the second-order neurons in the aforementioned nuclei decussate along the dorsal border of the *ml* and are, therefore, called the trigeminal lemniscus (Torvik 1957; Smith 1975; Matsushita et al. 1982). The function of the *vtt* is thought to be the conduction of vital sensory information, including nociceptive information. This study shows that the *vtt* indeed runs posterior to the *ml* and some imaging techniques even show that there is no clear distinction between the *ml* and the *vtt*. However, the high-resolution PLI images show that fibers of the *ml* and fibers of the *vtt* do not entwine along their courses.

The *dtt* has been described to consist of fibers originating from the dorsal part of the *PSN* and the caudal two-thirds of the *SN* (Burton and Craig 1979; Matsushita et al. 1982). It has been assumed that the *dtt* is responsible for conducting pain, temperature, and mechanoreceptive stimuli from the anterior part of the facial region and the intraoral cavity [for a review see (Henssen et al. 2016)].

This anatomical study provides further evidence of the existence of both the *dtt* and *vtt*; however, it cannot verify that both tracts are involved in the conduction of noxious stimuli. It is well known that the primary afferents of the somatosensory system can be subdivided according to myelinization and size. The different fiber types cluster the afferents into group A–C, of which the unmyelinated C-fibers and thin, myelinated Aδ-fibers subserve nociceptive transmission, whereas B-fibers represent thin preganglionic fibers of the autonomic nervous system (Erlanger and Gasser 1937). Central white matter, on the other hand, cannot be allocated to nociceptive systems on the basis of its architecture alone. For this reason, although the *dtt* seems to be present in humans as well, its function cannot be determined by this study.

### Technical considerations

The use of diffusion magnetic resonance imaging (dMRI) and tractography has become a popular method to study white matter pathways (Catani and Thiebaut de Schotten 2008). However, due to the limited imaging resolution of in vivo dMRI (typically 1.0–2.0 mm), its outcomes should be interpreted with care, especially in the presence of small fiber bundles. However, a recent study of Setsompop showed that on a clinical 3T scanner, imaging resolution of whole-brain data could be acquired of 0.66 mm and 0.76 mm isotropic. This indicates that new efficient methods that are capable of acquiring high-resolution diffusion data in vivo might be possible in the near future (Setsompop et al. 2018). Nevertheless, the applied MRI protocol in this study was found to further increase resolution to a spatial resolution of 0.2 mm isotropic for the T2*-images and 0.5 mm for the diffusion data. Although this resolution is higher than the resolution obtained in the study of Setsompop et al., it must be underlined that the present data was acquired on a preclinical scanning system, using 11.7T in an ex-vivo setting, whereas the setting of Setsompop et al. was a clinical 3T in vivo setting. One of the limitations of this study is formed by the fact that MR imaging parameters were not based on distribution of quantitative longitudinal relaxation time (T1) and transverse relaxation time (T2) and mean diffusivity (D) (Beaujoin et al. 2017, 2018). Therefore, corresponding histograms of T1, T2 and D values which could have been used to optimize the imaging protocol, are lacking from this study. On the other hand, an adapted protocol that came forth from empirical investigation (Kleinnijenhuis et al. 2013) was applied and yielded the aforementioned results. Furthermore, it is known that tractography can produce plausible-looking bundles in locations where the white matter tract does not actually exist, indicating that tractography produces substantial amounts of false-positive fiber bundles (Maier-Hein et al. 2017). To optimize the investigation of white matter microstructure, TDI was used as this technique uses reinterpolation of the quantitative maps at a finer resolution than the imaging resolution, which allows researchers to study white matter structure smaller than the imaging resolution (Calamante et al. 2010). As such, dMRI of ex vivo tissue allows for further evaluation of the tractography results by direct comparison against histological reference measures within the same specimen (Miller et al. 2011). Furthermore, complementation of the tractography data by use of PLI has been suggested to overcome the coarse sensitivity of diffusion tractography with respect to the identification of sharp bends, twists, and undulations (Foxley et al. 2016).

One of the limitations of this study is formed by the fact that the trigeminal tracts were not followed up to the thalamus due to the size of the coil of the 11.7T MR-scanner. Therefore, we cannot confirm that the *dtt* is connected to the thalamus as has been proposed in animal-based studies (Torvik 1957). Furthermore, the restricted number of specimens and the lack of other complementary neuroimaging techniques like optical coherence tomography (OCT) form another limitation of this study. OCT provides high-resolution fiber architecture data at approximately 1 micron spatial resolution. OCT is analogous to ultrasound techniques as it measures the backscattered light of a sample. Specifically, it is sensitive to variations of the refraction index in different types of tissue. For example, in 2014, Magnain and colleagues suggested that OCT can be used to generate accurate 3D reconstructions of histological layers in the cerebral cortex (Magnain et al. 2014). Furthermore, polarization-sensitive OCT is a special form of OCT that uses the intrinsic optical properties of back-scattering and birefringence of neural tissue. In this way, polarization-sensitive OCT has been used to image the fiber orientation and cyto- and myeloarchitecture of human nervous tissue and to visualize volumetric reconstructions of human brain tissue (Wang et al. 2018). 3D-PLI, however, also provides the possibility to investigate three-dimensional fiber orientations in sectioned tissue. Each section is measured separately and basically provides a 3D vector field description of fiber orientations, forming the basis for tractography (Axer et al. 2011c). Finally, this study did not use tracers, whereas most of the knowledge on which we base our neuroanatomical insights regarding the trigeminal connections are derived from animal-based tracer studies [for a review see (Henssen et al. 2016)]. In 2013, Seehaus et al. used fluorescent, retrograde tracers to stain projection fibers in human tissue, showing that tracing studies can be performed in a post-mortem setting on adult human neural tissue. However, the tracer was injected into the subcortical white matter (approximately 500–1000 µm from the cortex) of the upper part of the temporal lobe. After 48 months, the tracer had traced a tract with a maximal length of 13 mm, which makes it unsuitable for human tracing studies. The same study, however, reported that DTI is capable of reflecting the shape and orientation of nerve pathways (Seehaus et al. 2013).

Future research should investigate the functionality of the *vtt* and the *dtt* to determine the clinical significance of functional entities like the TSNC with respect to different trigeminothalamic tracts.

## Conclusion

This study shows that the *tt* bifurcates at the level of the human brainstem into two pathways which have previously been infrequently described in birds, reptiles, rodents, and non-human primates. These fiber tracts course in a ventral and dorsal direction and are, therefore, called the *vtt* and *dtt*, respectively. Connectivity, functionality, and clinical significance of these pathways must be determined in future studies.

## References

[CR1] Andersson JL, Sotiropoulos SN (2015). Non-parametric representation and prediction of single- and multi-shell diffusion-weighted MRI data using Gaussian processes. NeuroImage.

[CR2] Axer H, Beck S, Axer M, Schuchardt F, Heepe J, Flucken A, Axer M, Prescher A, Witte OW (2011). Microstructural analysis of human white matter architecture using polarized light imaging: views from neuroanatomy. Front Neuroinf.

[CR3] Axer M, Amunts K, Grassel D, Palm C, Dammers J, Axer H, Pietrzyk U, Zilles K (2011). A novel approach to the human connectome: ultra-high resolution mapping of fiber tracts in the brain. NeuroImage.

[CR4] Axer M, Grassel D, Kleiner M, Dammers J, Dickscheid T, Reckfort J, Hutz T, Eiben B, Pietrzyk U, Zilles K, Amunts K (2011). High-resolution fiber tract reconstruction in the human brain by means of three-dimensional polarized light imaging. Front Neuroinf.

[CR5] Beaujoin J, Destrieux C, Bernard J, Poupon F, Mangin JF, Poupon C (2017) Characterization of the brainstem connectivity and its microstructure using diffusion MR microscopy at ultra-high field (UHF) with strong gradients. Paper presented at the ISMRM 25th Annual Meeting Honolulu

[CR6] Beaujoin J, Palomero-Gallagher N, Boumezbeur F, Axer M, Bernard J, Poupon F, Schmitz D, Mangin JF, Poupon C (2018). Post-mortem inference of the human hippocampal connectivity and microstructure using ultra-high field diffusion MRI at 11.7 T. Brain Struct Funct.

[CR7] Behrens TE, Berg HJ, Jbabdi S, Rushworth MF, Woolrich MW (2007). Probabilistic diffusion tractography with multiple fibre orientations: what can we gain?. NeuroImage.

[CR8] Burton H, Craig AD (1979). Distribution of trigeminothalamic projection cells in cat and monkey. Brain Res.

[CR9] Büttner-Ennever JA, Horn AKE (2013). Olzewski and Baxter’s cytoarchitecture of the human brainstem.

[CR10] Calamante F, Tournier JD, Jackson GD, Connelly A (2010). Track-density imaging (TDI): super-resolution white matter imaging using whole-brain track-density mapping. NeuroImage.

[CR11] Calamante F, Tournier JD, Heidemann RM, Anwander A, Jackson GD, Connelly A (2011). Track density imaging (TDI): validation of super resolution property. NeuroImage.

[CR12] Catani M, Thiebaut de Schotten M (2008). A diffusion tensor imaging tractography atlas for virtual in vivo dissections. Cortex.

[CR13] D’Arceuil H, de Crespigny A (2007). The effects of brain tissue decomposition on diffusion tensor imaging and tractography. NeuroImage.

[CR14] Dyrby TB, Sogaard LV, Parker GJ, Alexander DC, Lind NM, Baare WF, Hay-Schmidt A, Eriksen N, Pakkenberg B, Paulson OB, Jelsing J (2007). Validation of in vitro probabilistic tractography. NeuroImage.

[CR15] Erlanger J, Gasser HS (1937). Electrical signs of nervous activity.

[CR16] Foxley S, Mollink J, Jbabdi S, Clare S, Hernandez Fernandez M, Scott C, Ansorge O, Miller KL (2016) Validating tractography of high resolution post-mortem human brain at 7T with polarized light imaging. Paper presented at the ISMRM 24th Annual Meeting Singapore

[CR17] Gonzalez-Rodriguez D, Guillou L, Cornat F, Lafaurie-Janvore J, Babataheri A, de Langre E, Barakat AI, Husson J (2016). Mechanical criterion for the rupture of a cell membrane under compression. Biophys J.

[CR18] Henssen DJ, Kurt E, Kozicz T, van Dongen R, Bartels RH, vanvan CappellenWalsum AM (2016). New insights in trigeminal anatomy: a double orofacial tract for nociceptive input. Front Neuroanat.

[CR19] Henssen DJHA, Kurt E, van Cappellen van Walsum AM, van Arnts I, Doorduin J (2018). Long-term effect of motor cortex stimulation in patients suffering from chronic neuropathic pain: an observational study. PLOS ONE.

[CR20] Holzapfel GA, Stadler M, Gasser TC (2005). Changes in the mechanical environment of stenotic arteries during interaction with stents: computational assessment of parametric stent designs. J Biomech Eng.

[CR21] Jantsch HH, Kemppainen P, Ringler R, Handwerker HO, Forster C (2005). Cortical representation of experimental tooth pain in humans. Pain.

[CR22] Jenkinson M, Beckmann CF, Behrens TE, Woolrich MW, Smith SM (2012). Fsl. NeuroImage.

[CR23] Kivrak BG, Erzurumlu RS (2013). Development of the principal nucleus trigeminal lemniscal projections in the mouse. J Comp Neurol.

[CR24] Kleinnijenhuis M, Zerbi V, Kusters B, Slump CH, Barth M, van Cappellen van Walsum AM (2013). Layer-specific diffusion weighted imaging in human primary visual cortex in vitro. Cortex.

[CR25] Magnain C, Augustinack JC, Reuter M, Wachinger C, Frosch MP, Ragan T, Akkin T, Wedeen VJ, Boas DA, Fischl B (2014). Blockface histology with optical coherence tomography: a comparison with Nissl staining. NeuroImage.

[CR26] Maier-Hein KH, Neher PF, Houde JC, Cote MA, Garyfallidis E, Zhong J, Chamberland M, Yeh FC, Lin YC, Ji Q, Reddick WE, Glass JO, Chen DQ, Feng Y, Gao C, Wu Y, Ma J, Renjie H, Li Q, Westin CF, Deslauriers-Gauthier S, Gonzalez JOO, Paquette M, St-Jean S, Girard G, Rheault F, Sidhu J, Tax CMW, Guo F, Mesri HY, David S, Froeling M, Heemskerk AM, Leemans A, Bore A, Pinsard B, Bedetti C, Desrosiers M, Brambati S, Doyon J, Sarica A, Vasta R, Cerasa A, Quattrone A, Yeatman J, Khan AR, Hodges W, Alexander S, Romascano D, Barakovic M, Auria A, Esteban O, Lemkaddem A, Thiran JP, Cetingul HE, Odry BL, Mailhe B, Nadar MS, Pizzagalli F, Prasad G, Villalon-Reina JE, Galvis J, Thompson PM, Requejo FS, Laguna PL, Lacerda LM, Barrett R, Dell’Acqua F, Catani M, Petit L, Caruyer E, Daducci A, Dyrby TB, Holland-Letz T, Hilgetag CC, Stieltjes B, Descoteaux M (2017). The challenge of mapping the human connectome based on diffusion tractography. Nat Commun.

[CR27] Matsushita M, Ikeda M, Okado N (1982). The cells of origin of the trigeminothalamic, trigeminospinal and trigeminocerebellar projections in the cat. Neuroscience.

[CR28] Miller KL, Stagg CJ, Douaud G, Jbabdi S, Smith SM, Behrens TE, Jenkinson M, Chance SA, Esiri MM, Voets NL, Jenkinson N, Aziz TZ, Turner MR, Johansen-Berg H, McNab JA (2011). Diffusion imaging of whole, post-mortem human brains on a clinical MRI scanner. NeuroImage.

[CR29] Nash PG, Macefield VG, Klineberg IJ, Murray GM, Henderson LA (2009). Differential activation of the human trigeminal nuclear complex by noxious and non-noxious orofacial stimulation. Hum Brain Mapp.

[CR30] Nash PG, Macefield VG, Klineberg IJ, Gustin SM, Murray GM, Henderson LA (2010). Bilateral activation of the trigeminothalamic tract by acute orofacial cutaneous and muscle pain in humans. Pain.

[CR31] Ralston HJ (2005). Pain and the primate thalamus. Prog Brain Res.

[CR32] Rausell E, Jones EG (1991). Chemically distinct compartments of the thalamic VPM nucleus in monkeys relay principal and spinal trigeminal pathways to different layers of the somatosensory cortex. J Neurosci.

[CR33] Rausell E, Jones EG (1991). Histochemical and immunocytochemical compartments of the thalamic VPM nucleus in monkeys and their relationship to the representational map. J Neurosci.

[CR34] Reckfort J, Wiese H, Pietrzyk U, Zilles K, Amunts K, Axer M (2015). A multiscale approach for the reconstruction of the fiber architecture of the human brain based on 3D-PLI. Front Neuroanat.

[CR35] Schmierer K, Wheeler-Kingshott CA, Tozer DJ, Boulby PA, Parkes HG, Yousry TA, Scaravilli F, Barker GJ, Tofts PS, Miller DH (2008). Quantitative magnetic resonance of postmortem multiple sclerosis brain before and after fixation. Magn Reson Med.

[CR36] Seehaus AK, Roebroeck A, Chiry O, Kim DS, Ronen I, Bratzke H, Goebel R, Galuske RA (2013). Histological validation of DW-MRI tractography in human postmortem tissue. Cereb Cortex.

[CR37] Sessle BJ (2000). Acute and chronic craniofacial pain: brainstem mechanisms of nociceptive transmission and neuroplasticity, and their clinical correlates. Crit Rev Oral Biol Med.

[CR38] Setsompop K, Fan Q, Stockmann J, Bilgic B, Huang S, Cauley SF, Nummenmaa A, Wang F, Rathi Y, Witzel T, Wald LL (2018). High-resolution in vivo diffusion imaging of the human brain with generalized slice dithered enhanced resolution: Simultaneous multislice (gSlider-SMS). Magn Reson Med.

[CR39] Shepherd TM, Thelwall PE, Stanisz GJ, Blackband SJ (2009). Aldehyde fixative solutions alter the water relaxation and diffusion properties of nervous tissue. Magn Reson Med.

[CR40] Smith RL (1975). Axonal projections and connections of the principal sensory trigeminal nucleus in the monkey. J Comp Neurol.

[CR41] Solstrand Dahlberg L, Linnman CN, Lee D, Burstein R, Becerra L, Borsook D (2018). Responsivity of periaqueductal gray connectivity is related to headache frequency in episodic migraine. Front Neurol.

[CR42] Stein BE, Price DD, Gazzaniga MS (1989). Pain perception in a man with total corpus callosum transection. Pain.

[CR43] Torvik A (1957). The ascending fibers from the main trigeminal sensory nucleus; an experimental study in the cat. Am J Anat.

[CR44] Wang H, Magnain C, Wang R, Dubb J, Varjabedian A, Tirrell LS, Stevens A, Augustinack JC, Konukoglu E, Aganj I, Frosch MP, Schmahmann JD, Fischl B, Boas DA (2018). as-PSOCT: volumetric microscopic imaging of human brain architecture and connectivity. NeuroImage.

